# Correction: *SELP* Asp603Asn and severe thrombosis in COVID-19 males

**DOI:** 10.1186/s13045-023-01415-7

**Published:** 2023-02-15

**Authors:** Chiara Fallerini, Sergio Daga, Elisa Benetti, Nicola Picchiotti, Kristina Zguro, Francesca Catapano, Virginia Baroni, Simone Lanini, Alessandro Bucalossi, Giuseppe Marotta, Francesca Colombo, Margherita Baldassarri, Francesca Fava, Giada Beligni, Laura Di Sarno, Diana Alaverdian, Maria Palmieri, Susanna Croci, Andrea M. Isidori, Simone Furini, Elisa Frullanti, on behalf of GEN-COVID Multicenter Study, Alessandra Renieri, Francesca Mari

**Affiliations:** 1 Medical Genetics Unit, University of Siena, Policlinico Le Scotte, Viale Bracci, 2, 53100 Siena, Italy; 2 Department of Medical Biotechnologies, Med Biotech Hub and Competence Center, University of Siena, Siena, Italy; 3 Department of Mathematics, University of Pavia, Pavia, Italy; 4 DIISM-SAILAB, University of Siena, Siena, Italy; 5 National Institute for the Infectious Diseases “L. Spallanzani”, Rome, Italy; 6 Stem Cell Transplant and Cellular Therapy Unit, University Hospital, Siena, Italy; 7 Istituto di Tecnologie Biomediche – Consiglio Nazionale delle Ricerche, Segrate, MI Italy; 8 Genetica Medica, Azienda Ospedaliero-Universitaria Senese, Siena, Italy; 9 Department of Experimental Medicine, Sapienza University of Rome, Rome, Italy

**Correction: J Hematol Oncol (2021) 14:123** https://doi.org/10.1186/s13045-021-01136-9

The original article [1] mistakenly omitted a sentence in the Acknowledgements section which the authors include ahead:

‘Two of several authors of this publication are members of the European Reference Network for rare haematological diseases EuroBloodNet.’.
